# **How many TCR clonotypes does a body maintain?**

**DOI:** 10.1016/j.jtbi.2015.10.016

**Published:** 2016-01-21

**Authors:** Grant Lythe, Robin E. Callard, Rollo L. Hoare, Carmen Molina-París

**Affiliations:** a Department of Applied Mathematics, School of Mathematics, University of Leeds, Leeds LS2 9JT, UK; b Institute for Child Health, University College London, 30 Guilford Street, London WC1N 1EH, UK; c Centre for Mathematics and Physics in the Life Sciences and Experimental Biology, University College London, Gower Street, London WC1N 1EH, UK

**Keywords:** T cells, Clonal repertoire, Homeostasis, Competition, Extinction, Stochastic modelling

## Abstract

We consider the lifetime of a T cell clonotype, the set of T cells with the same T cell receptor, from its thymic origin to its extinction in a multiclonal repertoire. Using published estimates of total cell numbers and thymic production rates, we calculate the mean number of cells per TCR clonotype, and the total number of clonotypes, in mice and humans. When there is little peripheral division, as in a mouse, the number of cells per clonotype is small and governed by the number of cells with identical TCR that exit the thymus. In humans, peripheral division is important and a clonotype may survive for decades, during which it expands to comprise many cells. We therefore devise and analyse a computational model of homeostasis of a multiclonal population. Each T cell in the model competes for self pMHC stimuli, cells of any one clonotype only recognising a small fraction of the many subsets of stimuli. A constant mean total number of cells is maintained by a balance between cell division and death, and a stable number of clonotypes by a balance between thymic production of new clonotypes and extinction of existing ones. The number of distinct clonotypes in a human body may be smaller than the total number of naive T cells by only one order of magnitude.

## Highlights

• The number of T cells of one clonotype is an integer.
• The history of a clonotype starts with release from the thymus, and ends with extinction.
• Competition and cross-reactivity are included in a natural way.
• The average number of cells per clonotype, in a human body, is only of order 10.

## Graphical abstract

**Figure:**
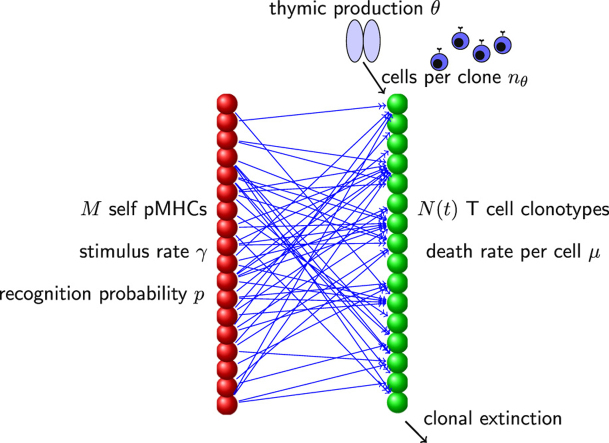

## 1 Introduction

Approximately $4\times 10^{11}$ T cells circulate in the adult human body (Jenkins et al., 2009), each with multiple T cell receptors (TCR) (Varma, 2008) on its surface. Each T cell is descended from a T cell that left the thymus after selection by binding to self-peptides expressed in association with major histocompatibility complex molecules (self pMHC) (Bains et al., 2009, Van Laethem et al., 2012), or is itself a thymic emigrant. The immune system can respond to very many different pathogens because very many different TCRs are present in the body. However, the TCRs present on the surface of one T cell are usually all identical. The set of cells with the same TCR defines a T cell clonotype, and the set of T cells in the body can be thought of as a repertoire of clonotypes. How many TCR clonotypes are there in humans, mice and other mammals? (Langman and Cohn, 1987, Blattman et al., 2002, Ciupe et al., 2013, Thomas et al., 2014). Estimates of the number of different TCRs that could, in principle, be produced by VDJ gene rearrangement in the thymus, are about 10^15^ (Sewell, 2012, Nikolich-Žugich et al., 2004, Zarnitsyna et al., 2013, Murugan et al., 2012). However, the human body cannot contain even one T cell of 10^15^ possible types: 10^15^ T cells would weigh about 500 kg (Mason, 1998).

Extracting and reverse transcribing RNA from pools of T cells, and amplifying, by PCR, the gene sequences that encode the TCR *β* chain, makes it possible to estimate the TCR diversity in a sample of blood. Initial estimates, based on extrapolation from a small fraction of the repertoire (Arstila et al., 1999), and more recent studies that are able to directly count large number of sequences and perform “missing species” analyses (Robins et al., 2009, Warren et al., 2011, Rempala and Seweryn, 2013), yield estimates of 10^6^ to 10^8^ (Qi et al., 2014). A new field of immunosequencing has emerged with technologies designed to sequence TCRs (Robins, 2013). Millions of TCR sequences can be amplified in a single multiplex PCR reaction, prepared and then read in parallel from a single sample. The distribution of gene usage can be measured with flow cytometry (Salameire et al., 2009, Ciupe et al., 2013), and used to track the dependence on phenotype, age and variation between individuals (Naylor et al., 2005, Britanova et al., 2014, Elhanati. et al., 2014, Becattini et al., 2015). Depending on the number of TCR*α* chains that each TCR*β* chain combines with, the number of distinct clonotypes in one human may be much higher than estimates based on TCR*β* alone (Keşmir et al., 2000). The spleen of a mouse has been estimated to contain $2\times 10^{6}$ clones of about 10 cells each (Casrouge et al., 2000). In mice, different T cell types can be compared and the effects of infections and immunization on the repertoire can be tracked (Bousso et al., 1998, Venturi et al., 2008, Bergot et al., 2015, Thomas et al., 2014).

In an adult, the number of recirculating T cells and the number of distinct clonotypes are believed to be held nearly constant for decades by balancing T cell loss with input from the thymus and homeostatic mechanisms controlling cell division and death in the periphery (Cannon, 1932, Tanchot et al., 1997, De Boer and Perelson, 1997, Goldrath and Bevan, 1999, Berzins et al., 2002, Murray et al., 2003, Troy and Shen, 2003, Seddon and Zamoyska, 2003, Takada and Jameson, 2009, Rudd et al., 2011, Germain, 2012). The diversity of the T cell repertoire in the periphery is made possible by the enormous variability of their self pMHC ligands (De Boer and Perelson, 1995, Moses et al., 2003, Blanchfield et al., 2013). Division of T cells in the periphery is determined by competition for stimuli from self-peptides, presented in association with MHC class I (for CD8^+^ T cells) and class II (for CD4^+^ T cells), found on antigen presenting cells in the lymph nodes, and by soluble factors including IL-7 for naive T cells and IL-15 for memory T cells.

Emerging from the thymus with a pattern of recognition of self pMHC that enabled it to survive positive and negative selection, each TCR clonotype is a species that competes for “space” or “niche” in the periphery (De Boer and Perelson, 1997, Tanchot et al., 1997, Goldrath and Bevan, 1999, Jameson, 2002, Troy and Shen, 2003, Hataye et al., 2006, Moon et al., 2007, Agenes et al., 2008, Leitao et al., 2009). Competition between cells of the same clonotype has been demonstrated by transfer of T cells to TCR transgenic hosts of differing or identical clonotype (Hataye et al., 2006, Min et al., 2004). These and other experiments suggest that, in lymphoreplete conditions, T cells compete for specific survival signals provided by TCR recognition of self pMHC ligands (Hataye et al., 2006, Min et al., 2004). Using pools of 100 T cells each, Singh et al. observed competition between T cell clonotypes for a shared self-ligand, each self pMHC being recognised only by T cells from a small fraction of clonotypes (Singh et al., 2012). Naive T cells in mice have lifetimes measured in months; nearly all are thymic emigrants who have not undergone peripheral division (den Braber et al., 2012, Thomas et al., 2013). Naive T cells in the human body have lifetimes measured in years; most naive peripheral T cells are daughter cells of other naive peripheral T cells (Vrisekoop et al., 2008, Murray et al., 2003, Houston et al., 2011).

The number of distinct TCR clonotypes, *N*, is equal to the total number of T cells divided by the mean number of cells per clonotype. Equivalently, *N* is equal to the product of the rate of release of new clonotypes from the thymus to the periphery, *θ*, and the mean lifetime of a clonotype in the periphery. We estimate the mean lifetime of a clonotype using analysis of a computational model with simple assumptions. Combined with estimates of the total number of T cells, this calculation yields estimates of the total number of clonotypes. The case of homeostasis in mice is rather simple: because there is little or no cell division in the periphery, the mean number of cells per clonotype is less than the mean number of cells, per clonotype, that emerge from the thymus. To understand homeostasis in the human body, however, we consider rates of thymic output, cell death and division, using published estimates and our model. We are able to find an explicit mathematical expression for the mean lifetime of a clonotype, and hence estimate the mean number of clonotypes.

In this paper, we develop a stochastic model of homeostasis of naive T cells, in which TCR signals and competition within the multiclonal T cell pool regulate the size and diversity of the naive T cell pool. We have in mind the homeostasis of naive αβ CD4^+^ human T cells, but the structure of the mathematical model is that of an ecological competition process involving many similar species (Merrill et al., 1994, De Boer and Perelson, 1995, De Boer and Perelson, 1997, Tanchot et al., 1997, Freitas and Rocha, 2000, Mahajan et al., 2005, Thomas-Vaslin et al., 2008). The number of individual cells in each species (here, TCR clonotype) changes due to cell death and division (De Boer and Perelson, 1997, Antia et al., 1998, Ciupe et al., 2009). In our model, the effect of the thymus is to create new clonotypes, not to add new cells to existing clonotypes. We have previously analysed models that focus on the dynamics of one or two clonotypes without explicitly taking the multiclonal nature of the TCR repertoire into account (Stirk et al., 2008, Stirk et al., 2010, Molina-París et al., 2011, Molina-París et al., 2011). In this work, the variables are the integer numbers of cells of each clonotype; a death or division event changes one of the variables by one cell. No clonotype has a pre-established self-limiting size; individual clonotypes compete for resources in a highly cross-reactive pattern of self pMHC recognition, which determines whether they die or survive. The fate of individual cells determines the fate of a clonotype and the fate of individual clonotypes determines the homeostasis of the repertoire. New clonotypes are produced by the thymus but we assume that the thymus does not produce cells of a pre-existing type, and that each individual T cell has only one type of TCR. On the other hand, division of T cells in the periphery can only contribute to the survival or expansion of existing clonotypes (Houston et al., 2011, den Braber et al., 2012, Johnson et al., 2012). Extinction of a clonotype, when it occurs, is thus irreversible.

### 1.1 Parameter values for humans and mice

The variables of the model are the numbers of cells of each clonotype; the parameters are the mean cell death rate, *μ*, the rate of cell division caused by stimulus from one self pMHC subset, *γ*, the rate at which new clonotypes are produced by the thymus, *θ*, the number of cells in a newly-produced clonotype, $n_{\theta }$, the total number of self pMHC subsets, *M*, and the probability that any given self pMHC is recognised by a randomly-selected T cell clonotype, *p*. In order to give a concrete estimate of clonotype numbers, we consider the values of *μ*, *γ*, *θ*, $n_{\theta }$, *M* and *p* that give an appropriate description of the naive CD4^+^ T cell repertoire in mice and humans.

The parameter *μ* sets the overall timescale that permits comparison of computational and physiological dynamics. We take *μ* to be constant, based on the simplifications that the T cell population is well mixed and that average availability of the IL-7 resource (Jameson, 2005, Bosco et al., 2005) is constant in time. The mean lifetime of a naive CD4^+^ T cell in the human body (Michie et al., 1992, Macallan et al., 2004, Borghans and De Boer, 2007) is 1–10 years (De Boer et al., 2012, De Boer et al., 2013, Westera et al., 2013, Westera et al., 2015). Thus, *μ* should be less than 1 year^−1^. In a mouse, the corresponding lifetime is about 1 month (Labrecque et al., 2001, Bourgeois et al., 2008, Westera et al., 2013); we take μ=1 month^−1^ when considering mice. Next, consider the total number of naive CD4^+^ T cells. A human body has about $2\times 10^{11}$; a mouse has about 10^7^ (Borghans and De Boer, 2007, Jenkins et al., 2009, Bains et al., 2009, den Braber et al., 2012). As we shall see, this number fixes the value of the combination γM/μ in humans, and constrains it in mice.

In our model, the number of subsets of pMHC stimuli is large, and individual TCRs are cross-reactive (Perelson and Oster, 1979, Evavold et al., 1995, Borghans et al., 1999, Newell et al., 2011, Calis and De Boer, 2012, van den Berg and Rand, 2007, Hao et al., 2006, Zarnitsyna et al., 2013, Yates, 2014, Birnbaum et al., 2014), recognising many different self pMHC; the number of subsets of self pMHC present is the parameter *M*. One estimate, based on the number of distinct 9-mers in the human proteome, is 10^7^ (Burroughs and de Boer, 2004). Another, based on the observation that peptides up to at least 14 amino acids long can be presented by MHC molecules, is as high as 10^16^ (Mason, 1998, Sewell, 2012). It may be more appropriate to count the different possible antigen presentation patterns on the surface of antigen presenting cells (Van Den Berg et al., 2001). The appropriate value of *M* is thus difficult to establish. Fortunately, our predictions are not very sensitive to the choice of *M*.

The total number of cells, and the rate of thymic production, differ from one type of mammal to another. On the other hand, the universe of different epitopes, presented as self pMHC to T cells, is a property of the mammal׳s environment, lifestyle and physiology. Patterns of cross-reactivity depend on molecular properties of T cell receptors and may depend on HLA class (Košmrlj et al., 2010). We take the value of *p*, the probability that interaction with a given self pMHC is capable of causing a randomly-selected T cell to divide, to be the same in humans and mice: $p=10^{-6}$ (Rizzuto et al., 2009, Su et al., 2013, Jenkins et al., 2009). Similarly, even though the body of a mouse is smaller than that of a human by close to a factor of 10^3^, we use the estimate $M=10^{10}$, in both cases. The number of cell divisions per unit time, in the whole body, produced by one self-pMHC subset, *γ*, is determined by the total amount of the resource available. We expect *γ* to be proportional to body weight, and thus larger in humans than in mice. The product γM is the total number of cell divisions per unit time in the periphery.

Thymic production rates have been estimated by direct and indirect methods (Scollay et al., 1980, Hazenberg et al., 2003, Thomas-Vaslin et al., 2008, Bains et al., 2009, den Braber et al., 2012). Because we seek to construct a model of homeostasis, we keep the parameter *θ* constant, although thymic output decreases with age in both mice and humans (Westera et al., 2015). The adult human thymus releases about 10^7^ naive CD4^+^ cells per day (den Braber et al., 2012), or $4\times 10^{9}$ cells per year. The thymus of a mouse produces about $4\times 10^{5}$ cells per day (den Braber et al., 2012), or 10^7^ per month. The last of our parameters, $n_{\theta }$, reflects the number of rounds of cell division in the thymus, after formation and selection of the full TCR but before a clonotype is released to the periphery (Hare et al., 1999, Hare et al., 2000, Sinclair et al., 2013, von Boehmer, 2014). For example, the choice $n_{\theta }=4$ corresponds to the assumption that a thymocyte undergoes two rounds of cell division, after expression and selection of the TCR but before exit from the thymus.

## 2 Results

Numerical realisations update the variables

$$n_{i}(t)=\mathrm{number}\;\mathrm{of}\;T\;\mathrm{cells}\;\mathrm{of}\;\mathrm{clonotype}\;i\;\mathrm{at}\;\mathrm{time}\;t.$$

A numerical realisation starts with N(0) clonotypes, when the index *i* that labels a clonotype runs from 1 to N(0). We shall be particularly interested in

N(t)=numberofsurvivingclonotypesattimet.

That is, *N*(*t*) is the number of *i* such that $n_{i}(t)>0$. In any short time interval, a T cell either stays alive, dies, or divides into two cells of the same clonotype. The dynamics are governed by the birth (division) and death rates. For the latter, we adopt the simplest hypothesis: that each T cell, regardless of its clonotype, has probability *μ* per unit time of dying. In the absence of cell division, the number of cells in a population would decrease following an exponential decay curve (Tanchot et al., 1997). The probability per unit time of a T cell undergoing division into two daughter cells of the same clonotype, is proportional to the stimulus that it receives from the set of self pMHCs. Our assumption for cell division is that the stimulus from each subset is divided equally on average between the set of all T cells that recognise it, a fraction of all T cells that comprises T cells of many different clonotypes.

The rates of cell division are calculated from their interactions with *M* self pMHCs by generating, at the start of the run, the N(0)×M matrix *A* with entries (Košmrlj et al., 2010, Molina-París et al., 2011):

(1) $$A_{\mathrm{iq}}=\{\begin{array}{cc} 1 & \mathrm{if}\;\mathrm{self}\;\mathrm{pMHC}\;q\;\mathrm{stimulates}\;T\;\mathrm{cells}\;\mathrm{of}\;\mathrm{clonotype}\;i\\ 0 & \mathrm{otherwise}. \end{array}$$

**Fig. 1:**
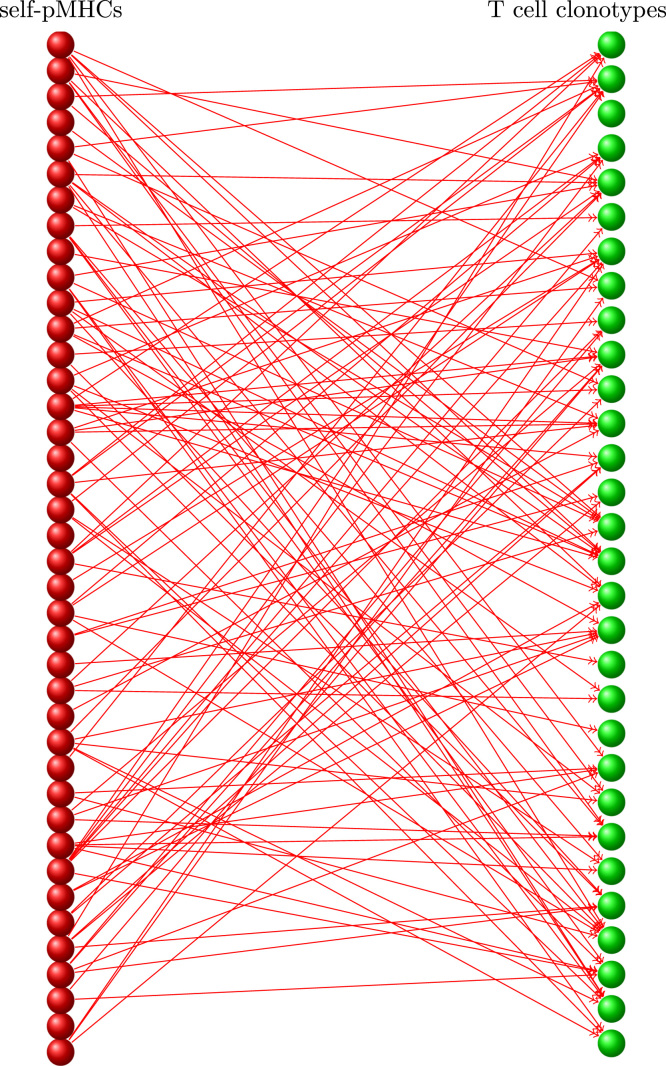
Representation of connections between *N*=30 T cell clonotypes and *M*=40 self pMHC subsets. An arrow connecting a red ball to a green ball indicates that the self pMHC stimulates division of cells in the T cell clonotype. The connections between clonotypes and self pMHCs are assigned randomly with probability *p*=0.1. That is, each entry of the matrix *A*, independently, is equal to 1 with probability *p* and equal to 0 with probability 1−p. (For interpretation of the references to color in this figure caption, the reader is referred to the web version of this paper.)

If $A_{\mathrm{iq}}=1$ then we say “self pMHC *q* stimulates T cells of clonotype *i* to divide” or, equivalently, “T cells of clonotype *i* recognise self pMHC *q*”. In graph theory, the matrix *A* is known as the biadjacency matrix of the bipartite graph consisting of the set of T cell clonotypes, the set of self pMHC and the set of connections (Asratian et al., 1998). A small-scale example is depicted in Fig. 1.

The characteristic signature of a T cell clonotype is its pattern of interaction with the set of *M* self-pMHC stimuli, illustrated in Fig. 1. Each of the self pMHC is recognised with probability *p* (Borghans et al., 1999) so that the average number of self pMHCs recognised by a TCR (cross-reactivity) is *pM*. The number of possible patterns is thus at least as large as the number of ways of choosing *pM* samples from a total of *M* possibilities. If *M*=1000 and *p*=0.1 then the probability that two clonotypes have the same pattern, given by ${\left({\left(1-p\right)}^{2}+p^{2}\right)}^{M}$, is already less than 10^−85^. The recognition pattern of any one clonotype is highly unlikely to ever be repeated and every clonotype, existing in the periphery or emerging from the thymus, may be taken as unique. Similarly, the repertoires of different individual mammals from the same species will be independent samples from the set of possibilities, with little overlap (Bousso et al., 1998).

The number of T cells recognising self pMHC *q* at time *t*, *c*_*q*_(*t*), and the division rate of clonotype *i*, $\Lambda_{i}(t)$, are given by

(2) $$c_{q}(t)=\overset{N(t)}{\underset{i=1}{\sum }}A_{\mathrm{iq}}n_{i}(t)\;\mathrm{and}\;\Lambda_{i}(t)=n_{i}(t)\overset{M}{\underset{q=1}{\sum }}\gamma_{q}A_{\mathrm{iq}}\frac{1}{c_{q}(t)},$$

where *γ*_*q*_ is the stimulus rate of self pMHC *q*. In this paper, we consider the case where all self pMHC deliver stimulus at the same rate, and the case where the rates are drawn from a log-normal distribution (see Methods). As Δt→0, $\Lambda_{i}(t)\Delta t$ is the probability that $n_{i}(t+\Delta t)-n_{i}(t)=1$, corresponding to one round of cell division in one of the T cells of clonotype *i* between *t* and t+Δt. The quantity $\frac{n_{i}(t)}{c_{q}(t)}$ is the share of the total stimulus from *q* that is received by clonotype *i*, because of the assumption that each self pMHC distributes its stimulus, with equal probability, amongst all T cells capable of receiving it. The dynamics of the *n*_*i*_(*t*) are coupled because each $\Lambda_{i}(t)$ depends on *n*_*j*_(*t*), for all clonotypes *j* that share a self pMHC with *i*.

In our model, the thymus is a source of new T cell specificities, whereas division of T cells in the periphery can only contribute to the survival or expansion of existing clonotypes (den Braber et al., 2012). New clonotypes are produced with rate *θ* and number of cells $n_{\theta }$. That is, with probability θΔt in any small time interval Δt, a new clonotype is defined, by drawing a subset of connections to the *M* self pMHCs, allocated $n_{\theta }$ cells, and added to the system. Characterised by their self pMHC recognition pattern, recent thymic emigrants (Berzins et al., 1999, Fink, 2013) in our model are not given any special advantage or disadvantage when competing with peripheral cells, although both possibilities have been suggested (Vrisekoop et al., 2008, Thomas-Vaslin et al., 2008, Houston et al., 2011).

### 2.1 Mean total number of cells

One immediate consequence of the structure of the competition model is that there is a well-defined homeostatic mean number of cells. Let the total number of T cells at time *t* be

$$n(t)=\overset{N(t)}{\underset{i=1}{\sum }}n_{i}(t)$$

and the mean total number of T cells at time *t* be x(t)=IE(n(t)). Note that *x*(*t*), the mean over realisations of the stochastic processes of the model, is not constrained to be an integer and satisfies the ordinary differential equation

(3) $$\frac{d}{dt}x=-\mu x+\Phi +\theta n_{\theta },$$

where $\Phi =\sum_{i=1}^{N(t)}\Lambda_{i}(t)$ is the total rate of production of new cells by peripheral division. As long as each of the *M* self pMHCs is recognised by at least one cell,

$$\Phi =\underset{q}{\sum }\gamma_{q}$$

is constant and

$$x(t)=(\Phi +\theta n_{\theta })\mu^{-1}(1-e^{-\mu t})+x(0)e^{-\mu t},$$

so that $x(t)\to \frac{\Phi +\theta n_{\theta }}{\mu }$, after a transient time of duration proportional to *μ*^−1^. In the case where *γ*_*q*_ is equal to *γ* for every *q*, Φ=γM and the stationary mean total number of cells is *x*^⁎^, where

(4) $$x^{⁎}=\frac{\gamma M+\theta n_{\theta }}{\mu }.$$

Note that this stably-maintained mean number of cells (De Boer and Perelson, 1997) arises as a global effect of balancing stochastic influences, but is not a unique state in the multi-dimensional space of clonal sizes. Indeed, as we shall see, individual clonotypes continue to follow their dynamics, reacting to changes in competing clonotypes and chance events, which can lead to extinction or successful establishment in the periphery.

The steady-state fraction, *r*, of cells that are thymic emigrants that have not divided in the periphery is given by $r=\theta n_{\theta }/(\gamma M+\theta n_{\theta })=\theta n_{\theta }/(\mu n(t))$ (den Braber et al., 2012). If division of peripheral cells is the dominant contribution, the mean total number of cells is approximately equal to γM/μ. In adult humans, therefore, if μ=0.5 year^−1^ and the total number of naive CD4^+^ T cells is $2\times 10^{11}$ then $\gamma M=10^{11}$ year^−1^. The observation that thymic production, rather than peripheral division, dominates the dynamics of murine T cell homeostasis implies that $\gamma M⪡10^{7}$, in a mouse. Assuming that the values of *M* are similar in humans and mice, it is necessary to assume that the ratio γ/μ is smaller in a mouse than in a human, by a factor which reflects the overall size of the pMHC resource available, proportional to body weight (Table 1).

### 2.2 How many TCR clonotypes does a body maintain?

We can make estimates of the stable number of clonotypes, *N*^⁎^, from estimates of the number of cells per clonotype. The dynamics in the “murine” limit is in fact rather simple: clonotypes operate independently, decay stochastically, and comprise few cells. New clonotypes, exported from the thymus, appear in the periphery with rate *θ*, initially consisting of $n_{\theta }$ cells. Each cell has a constant probability per unit time of dying but cell division is very unlikely. If $n_{\theta }=1$, then nearly every clonotype has only one cell. If not, the mean number of cells per clonotype is less than $n_{\theta }$. With the simple estimate that the mean number of cells per clonotype, averaged over the lifetime of the clonotype, is $n_{\theta }/2$, we find that the mean number of clonotypes in the body of a mouse is related to the mean number of naive T cells in the mouse, *x*, as t→∞, by

(5) $$N^{⁎}≃\frac{2}{n_{\theta }}x.$$

If we choose $n_{\theta }=4$ and $x≃10^{7}$, then we find $N^{⁎}≃5\times 10^{6}$.

We introduce the parameter

(6) $$\alpha =\frac{n_{\theta }\theta }{\gamma M}.$$

**Fig. 2:**
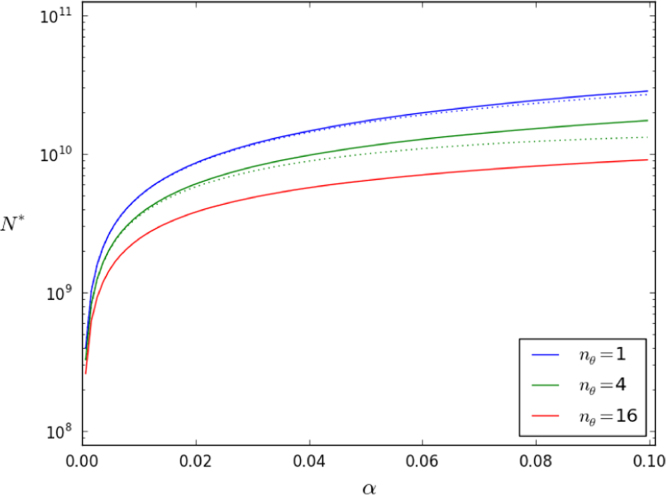
Predicted steady-state number of distinct clonotypes. The value of *N*^⁎^ is the product of the rate of production of new clonotypes, *θ*, and the mean lifetime of a clonotype, (16). The dotted lines are (7), valid in the weak-thymus limit. The parameter $\alpha =\frac{n_{\theta }\theta }{\gamma M}$ measures the strength of thymic production relative to peripheral division. Three values of $n_{\theta }$ are shown. We use $\gamma M/\mu =10^{11}$ cells, approximately equal to the number of naive CD4^+^ T cells in a human.

The relation (5) is valid in the weak peripheral division limit, α→0, appropriate to describe T cell homeostasis in a mouse. In the human body, on the other hand, many fewer cells result from thymic production than from peripheral division (Borghans and Tesselaar, 2009, den Braber et al., 2012). The stationary mean number of clonotypes in the periphery is found by multiplying the mean lifetime by the rate of production of new clonotypes, *θ*. In Methods, we compute the mean number of clonotypes by calculating the mean lifetime of a clonotype in the limit α⪡1:

(7) $$N^{⁎}\to \frac{\gamma M}{\mu }\alpha (1-\gamma_{E}-\log(n_{\theta }\alpha )),\;\mathrm{as}\;\alpha n_{\theta }\to 0,$$

where $\gamma_{E}=0.577\ldots$ is the Euler–Mascheroni constant. Recall that $\frac{\gamma M}{\mu }$ is the mean total number of cells. In Fig. 2, the solid lines use (16) and the dotted line is (7).

Thymic export is estimated to contribute less than 10 percent of the total production of new T cells in adults (den Braber et al., 2012). Let us identify where on the curve in Fig. 2, to locate naive CD4^+^ T cell homeostasis in adult humans. We use the estimate of annual production of cells by the thymus of $4\times 10^{9}$, and the estimate of annual number of peripheral divisions of 10^11^, to obtain $\frac{\theta n_{\theta }}{\gamma M}≃0.04$. Thus, we find that the number of TCR clonotypes in the body is nine percent of the corresponding total number of T cells in the body, close to 10^10^. In other words, our model of competition produces a mean clonal size, over the lifetime of a clone, that is only of order 10, even though some clonotypes expand to thousands of cells.

In Table 1, we collect our estimated values of the parameters. Based on these estimates, the number of distinct clonotypes in the human body is about ten times smaller than the total number of naive T cells $\left(N≃10^{10}\right)$ and the number of distinct clonotypes in a mouse is about two times smaller than the total number of naive T cells $\left(N≃10^{7}\right)$. These estimates are based on considering the lifetime of the population of cells making up one clonotype, starting with emergence from the thymus and ending in extinction in the periphery. Without peripheral division, the number of cells of one type does not exceed its initial value and the mean number of cells, averaged over the lifetime of the clonotype, is less than the number produced by the thymus, $n_{\theta }$. When there is peripheral division, the number of cells of a given clonotype may be large at some points in the lifetime of the clone, but the average over the lifetime of the clonotype is less than the maximum.

**Fig. 3:**
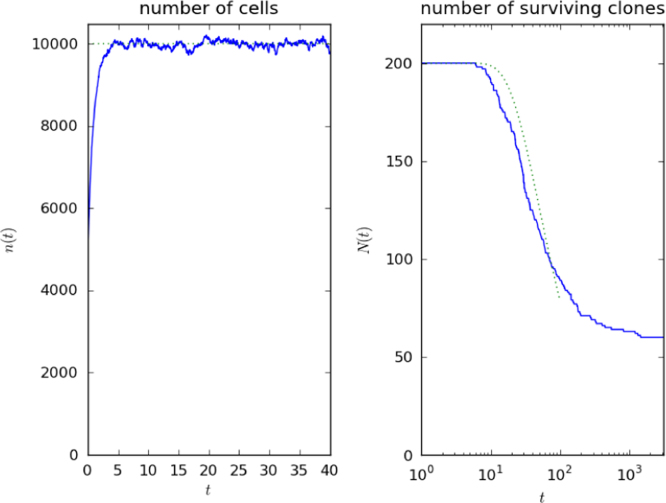
A numerical solution of the competition model without thymic production. The left panel shows the total number of T cells, as a function of time. (The total number of cells continues to fluctuate about the same mean for times later than shown.) The right panel shows the number of surviving clonotypes, *N*(*t*), with a logarithmic time scale. The dotted line is (14). One time unit is the mean lifetime of a T cell in the absence of division stimulus, taken to be one year in a human body. The parameters are μ=1.0, γ=10, *M*=1000; T cell clonotype-pMHC connections were assigned randomly with probability *p*=0.1 at the beginning of the run. The initial conditions are N(0)=200 and $n_{i}(0)=25$ for each *i*.

In Methods, we describe the implementation of the Gillespie algorithm to produce numerical realisations of the model. In Fig. 3, Fig. 5, Fig. 6, we display results from small-scale numerical realisations, without thymic input, that illustrate the extinction of clonotypes due to competition. Larger systems, maintaining an invariance from the perspective of any one clonotype, are shown in Fig. 7. The effect of thymic production is considered in Fig. 8. In the numerical realisations summarised in Fig. 9, each self pMHC is assigned a strength *γ*_*q*_ that is nor constant but drawn from a log-normal distribution. In Section 4.3, we consider the extinction time of the *i*th clonotype as a random variable, and derive an expression for its probability distribution using a diffusion approximation.

## 3 Discussion

Emerging from the thymus with a pattern of recognition of self pMHC, each TCR clonotype is a species that competes in the pool of pre-established ones. We have analysed naive T cell homeostasis by considering the lifetimes of sets of cells making up TCR clonotypes, from generation in the thymus to extinction in the periphery. Our point of view is to consider the stochastic dynamics of the number of T cells of one clonotype as a function of time, which increases or decreases according to the rates of cell division and death. Any one clonotype׳s time to extinction is a random variable that depends on its cross-reactivity, on the properties of those clonotypes with which it shares self-pMHC stimuli, and on average properties of the whole naive T cell repertoire. The model presented here has both interclonal and intraclonal competition, in which extinction of a clonotype is irreversible. By considering distributions of times to clonal extinction, we give estimates of clonal sizes, and hence diversity, in adult mice and humans. With the estimate that the ratio of thymic production to peripheral division of naive CD4^+^ T cells is four percent, our model predicts that the number of distinct clonotypes in the human body is nine percent of the total number of naive CD4^+^ T cells.

Our scheme is consistent with the idea that homeostasis of naive T cell numbers is maintained by availability of cytokines, especially IL-7 (Seddon et al., 2003, Min et al., 2004, Fry and Mackall, 2005, Tan et al., 2001, Pearson et al., 2011, Palmer et al., 2011, Koenen et al., 2013), that are available to T cells of all clonotypes (Ciupe et al., 2009), whereas the diversity of T cells is dependent on the varied self pMHC presented by the body׳s antigen presenting cells; each self pMHC being recognised only by T cells from a small fraction of clonotypes (Mahajan et al., 2005, Ciupe et al., 2009, Singh et al., 2012). It is possible to produce deterministic models of competition between clonotypes for different resources, with a biadjacency matrix, based on one ODE for each clonotype (De Boer and Perelson, 1997). Stochastic models have the virtue of being able to include chance, as well as systematic competition effects, of being accurate when the numbers of cells of any type are small, and of describing extinction of clonotypes without extra assumptions.

Producing a large-scale competition model with the simplest assumptions and minimum number of parameters comes, of course, at the cost of simplifying the dynamics of homeostasis of the immune system. There are a number of ways that the simple model used here can be changed to more closely resemble reality. One that we have already explored is to relax the assumption that all self-pMHC complexes provide stimuli that produce cell division with the same rate. Instead, we assigned strengths to each drawn from a log-normal distribution. Even when the variance was larger than the mean, the results were similar to the constant-*γ* case. We believe that it is desirable to include interclonal and intraclonal heterogeneity (Sinclair et al., 2011, Mandl et al., 2013) to allow exploration of post-thymic maturation (Thomas-Vaslin et al., 2008), of the effects of cell-to-cell differences manifested in variation of CD25, IL-7R and CD5 expression levels, and differentiation into distinct phenotypes at the single-cell level (Zehn et al., 2012, Hataye et al., 2006, Jenkins et al., 2009, Pepper and Jenkins, 2011). Changes over time leading to increase or decrease of division rates in some clonotypes (Johnson et al., 2012) may also occur. Our numerical realisations have used the exact Gillespie algorithm, without resort to approximations based on averaging over populations. In order to undertake direct larger-scale simulations, with numbers of cells closer to physiological values, methods that do not update the *Λ_i_* at every step, or efficient approximations such as “tau-leaping” (Márquez-Lago and Burrage, 2007), will be appropriate.

Although the model is designed to describe the long-term dynamics associated with naive T cell homeostasis, we envisage including strong but short-term stimuli (Hershberg et al., 2003) in the same framework to understand the effect of foreign antigen, the breadth of the immune response to a mutating virus (van Deutekom et al., 2013), and the creation of populations of memory cells, capable of persisting without TCR signals (Seddon and Zamoyska, 2003, Ganusov et al., 2006, Sprent and Surh, 2011). It is also necessary to consider the circulation of T cells around the body (Ganusov and Auerbach, 2014) and the distribution of different T cell subsets in different organs (Thome et al., 2014, Farber et al., 2014).

Dramatic illustrations of the consequences of perturbing homeostatic processes in the peripheral immune system are found in clinical or experimentally-induced lymphopenic environments (Bosco et al., 2005, Hogan et al., 2013, Paul et al., 2013). We can reproduce the reconstitution of the repertoire by thymic output and peripheral division with the model as presented here, but the constant-death-rate assumption will need to be modified to take into account the abundance of trophic factors during recovery from lymphopenia and heterogeneity in TCR and IL-7R expression levels (Palmer et al., 2011, Singh et al., 2012). Another interesting scenario is that of post-thymic transplant dynamics in infants with DiGeorge anomaly, where thymic production is explicitly a function of time (Freitas and Rocha, 2000, Houston et al., 2011, Ciupe et al., 2009).

## 4 Methods

### 4.1 The stochastic algorithm

At each step of the Gillespie algorithm (Renshaw, 2011, Wilkinson, 2006), one of the 2N(t)+1 possible events is chosen. The event is either death of a cell in some clonotype, *i*, chosen with probability $\mu n_{i}(t)/S(t)$, birth of a cell in clonotype *i*, chosen with probability $\Lambda_{i}(t)/S(t)$, or thymic production of a new clonotype, chosen with probability θ/S(t). Here, *S*(*t*) is the sum of the rates at time *t*:

$$S(t)=\overset{N(t)}{\underset{i}{\sum }}(\mu n_{i}(t)+\Lambda_{i}(t)+\theta ).$$

At the end of the step, time is incremented by an amount sampled from the exponential distribution with mean 1/S(t) (Wilkinson, 2006).

### 4.2 Competition

In Fig. 3, we display results from one small-scale numerical realisation, with θ=0 for simplicity. The effect of competition for stimulus is to drive some clonotypes to extinction, reducing the number of surviving clonotypes (Molina-París et al., 2011). The parameters are *p*=0.1 and $\gamma_{q}=10$ for every *q*. With the choice μ=1, one time unit is the mean lifetime of a T cell in the absence of stimulus (Borghans and De Boer, 2007, den Braber et al., 2012, De Boer et al., 2013). In this work, when we consider the cases of T cell homeostasis in humans and mice, we shall use the values μ=1 month^−1^ (mouse) (Thomas-Vaslin et al., 2008) and μ=1 year^−1^ (human) (Macallan et al., 2004).

**Fig. 4:**
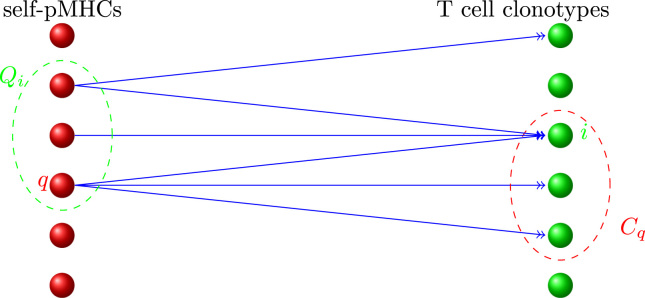
Diagrammatic representation of the competition model. Each green ball represents a set of T cells; each red ball, a set of self pMHC complexes. An arrow (link) connecting a red ball to a green indicates that T cells of the clonotype recognise the self pMHC subset. (For interpretation of the references to color in this figure caption, the reader is referred to the web version of this paper.)

The relationship between the set of clonotypes and the set of self pMHCs is illustrated in Fig. 1, Fig. 4. Consider the following quantities (Stirk et al., 2008, Stirk et al., 2010, Molina-París et al., 2011, Molina-París et al., 2011):

• The set of self pMHCs that are recognised by T cells of clonotype *i* is denoted by *Q*_*i*_, and the number of self pMHC subsets in *Q*_*i*_, by *ϕ*_*i*_.
• The set of T cells that recognise a self pMHC *q* is denoted by *C*_*q*_(*t*), and the number of surviving clonotypes in *C*_*q*_ by $\left|C_{q}\right|$.
• The average of *ϕ*_*i*_ over all surviving clonotypes is denoted $\overset{¯}{\phi }(t)$, and the average of $\left|C_{q}\right|$ over all *q* is denoted $\overset{¯}{C}(t)$ (Stirk et al., 2010).

An important exact relationship is (Mason, 1998, Zarnitsyna et al., 2013):

(8) $$M\overset{¯}{C}(t)=N(t)\overset{¯}{\phi }(t),$$

which holds because the LHS and RHS are, in the representation of Fig. 1, different ways of counting the total number of connections between T cell clonotypes and self pMHCs. In our model, *M* is constant in time; the remaining three quantities in (8) vary in time but the exact equality is maintained.

There are many ways to assign connections between clonotypes and self pMHCs. A simple algorithm, used in this work, is to assign all connections independently with probability *p*. The values of *ϕ*_*i*_ are thus samples from the binomial distribution with mean *pM*, and

$$\overset{¯}{\phi }(0)=\mathrm{pM}.$$

For sufficiently large *pM*, the distribution of values of *ϕ*_*i*_ is approximately Gaussian with standard deviation $\sqrt{\mathrm{pM}}$. Despite its simplicity, the assignment of connections to clonotypes reproduces the basic features of positive and negative thymic selection: all clonotypes recognise some self pMHC, but none are too specific or too promiscuous. 

**Fig. 5:**
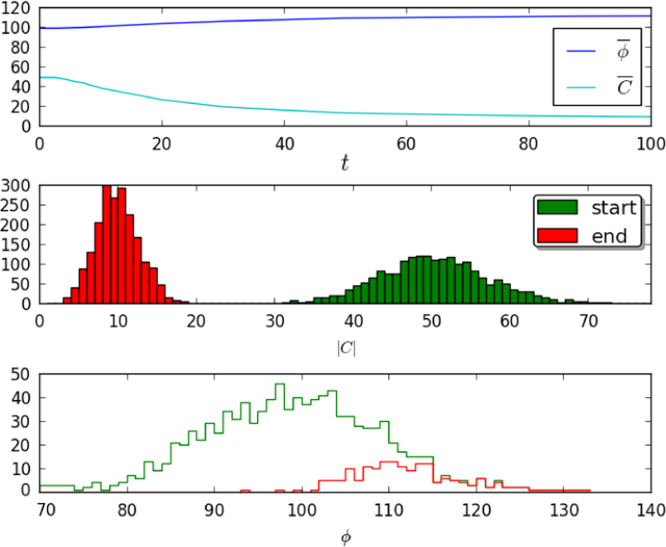
A numerical solution of the exact clonal competition model without thymic input. In the top panel, the average number of self pMHCs recognised by a clonotype, $\overset{¯}{\phi }(t)$, and the average number of clonotypes recognising a pMHC, $\overset{¯}{C}(t)$, are shown as a function of time. Due to extinction of clonotypes, $\overset{¯}{\phi }(t)$ increases and $\overset{¯}{C}(t)$ decreases. The middle panel shows histograms of the 2000 values of $\left|C_{q}\right|$, at the start and end of the realisation. The bottom panel shows the histograms of values of *ϕ* at the start (1000 clonotypes) and end (168 clonotypes). T cell clonotype-pMHC connections are assigned randomly with probability *p*=0.05. The remaining parameter values are μ=1.0, γ=10, *M*=2000. The initial conditions are N(0)=1000 and $n_{i}(0)=10\;\forall i$.

Fig. 5 illustrates the effect of extinction of clonotypes on mean quantities, averaged over the repertoire. Extinction increases $\overset{¯}{\phi }(t)$, which is an average over the surviving clonotypes, but the change is necessarily confined within the distribution of values of *ϕ*_*i*_ (mean *pM*, standard deviation $\sqrt{\mathrm{pM}}$). On the other hand, the average number of distinct clonotypes recognising a given self pMHC, $\overset{¯}{C}(t)$, is reduced because of extinction of clonotypes. As time increases, in the absence of thymic input, *N*(*t*) decreases, $\overset{¯}{\phi }(t)$ is nearly constant, and $\overset{¯}{C}(t)$ decreases in such a way that the equality (8) is maintained.

### 4.3 Timescales of clonal extinction

Our point of view is to consider the stochastic dynamics of the number of T cells of one clonotype as a function of time. Using the description of the dynamics of a typical clonotype in the periphery, we can find an expression for the distribution of its time to extinction. Let the extinction time of the *i*th clonotype be the random variable *τ*_*i*_. The number of cells, *n*_*i*_(*t*), of the clonotype with label *i* is an integer, increasing or decreasing by one cell at a time, according to the rates for cell division (“birth”) and death; thus *τ*_*i*_, the smallest value of *t* such that $n_{i}(t)=0$. The death rate is $\mu n_{i}(t)$, independent of the other clonotypes in the system. In contrast, the division rate, $\Lambda_{i}(t)$, depends on the number of cells of all clonotypes that compete for any of the self pMHC recognised by clonotype *i*. If we begin with the expression, (2), and replace the sums by their mean values so that $c_{q}(t)\to {\mathrm{px}}^{⁎}$ and $\sum_{q=1}^{M}\gamma_{q}A_{\mathrm{iq}}\to \gamma \mathrm{pM}$, then

(9) $$\Lambda_{i}(t)=n_{i}(t)\frac{\gamma \mathrm{pM}}{{\mathrm{px}}^{⁎}}=\frac{1}{1+\alpha }\mu n_{i}(t).$$

The most remarkable feature of (9) is that the birth rate $\Lambda_{i}(t)$ takes a form that is similar to the death rate, $\mu n_{i}(t)$. In the absence of thymic production,

(10) $$\Lambda_{i}(t)≃\mu n_{i}(t).$$

Although it is not surprising that death rates and birth rates are in balance for a typical clonotype established in the periphery, it is remarkable that they can take identical functional forms.

To derive an expression for the distribution of *τ*_*i*_, we use the diffusion approximation, where *n*_*i*_(*t*) is replaced by a diffusion process on the real line, $X_{t}$ (Taylor and Karlin, 1998, Renshaw, 2011). The equation of motion of $X_{t}$ is a stochastic differential equation (SDE) that is characterised by the mean and mean-square of its increments over a small time interval Δt (Gardiner, 2004, Wilkinson, 2006). Thus we must consider the mean change in the size of the population *n*_*i*_ between *t* and t+Δt:

(11) $$\mathrm{IE}(n_{i}(t+\Delta t))=n_{i}(t)+(\Lambda_{i}(t)-\mu n_{i}(t))\Delta t.$$

We require

$$\mathrm{IE}(X_{t+\Delta t}-X_{t})=-\frac{\alpha }{1+\alpha }\mu X_{t}\Delta t\;\mathrm{and}\;\mathrm{IE}({\left(X_{t+\Delta t}-X_{t}\right)}^{2})=\frac{2+\alpha }{1+\alpha }\mu X_{t}\Delta t.$$

Hence the stochastic differential equation is

(12) $$dX_{t}=-\mu \frac{\alpha }{1+\alpha }dt+\sqrt{\frac{2+\alpha }{1+\alpha }}\sqrt{\mu X_{t}}dW_{t},$$

with initial condition $X_{0}=b$.

A simple expression is found when α=0: the distribution of times at which the diffusion process reaches the absorbing state at 0 is Revuz and Yor (2004):

(13) $$P[X_{t}=0|X_{0}=b]=\exp\;(-\frac{b}{\mu t}).$$

The appropriate initial condition *b* is the mean total number of cells, γM/μ, divided by the initial number of clonotypes, N(0). That is, the probability that a randomly-chosen clonotype is extinct before time *t*, without thymic production, is

(14) $$P[\mathrm{extinction}]=\exp\;(-\frac{\gamma M}{N(0)\mu^{2}t}).$$

The relation (14) is used to plot the dotted lines in Fig. 3.

Now let us consider 0<α⪡1. That is, we consider non-zero thymic production, but where more cells are created by peripheral division than by the thymus. Then

(15) $$dX_{t}=-\alpha \mu X_{t}dX_{t}+\sqrt{2\mu X_{t}}dW_{t}.$$

The mean lifetime of a clonotype is the mean time until $X_{t}$ reaches zero, starting at *b*, T(α,b). It satisfies

$$\mu b\frac{d^{2}}{db^{2}}T(\alpha ,b)-\alpha \mu b\frac{d}{db}T(\alpha ,b)=-1.$$

The solution, with initial number of cells $n_{\theta }$, is

(16) $$T(\alpha ,n_{\theta })=\frac{1}{\alpha \mu }(\gamma_{E}-e^{\alpha n_{\theta }}\;\mathrm{Ei}\;(-\alpha n_{\theta })+\log(\alpha n_{\theta })),$$

where $\gamma_{E}=0.577\ldots$ is the Euler–Mascheroni constant. The expression (7) is obtained by using the properties of the exponential integral, Ei, for small values of its argument: as $\alpha n_{\theta }\to 0$, $\mathrm{Ei}(-\alpha n_{\theta })\to \gamma_{E}+\log(\alpha n_{\theta })-\alpha n_{\theta }$ and so $T(\alpha ,n_{\theta })\to \frac{n_{\theta }}{\mu }(1-\gamma_{E}-\log(\alpha n_{\theta }))$.

### 4.4 Selective or stochastic competition

**Fig. 6:**
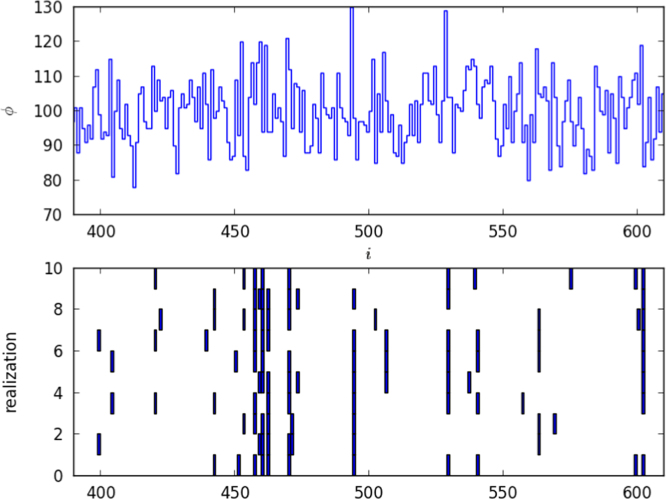
Competition is more important than chance in determining which clonotypes survive. Of the initial 1000 clonotypes, on average only 50 survive. Ten independent realisations were carried out with the same connection matrix *A*; the fates of clonotypes *i*=390 to *i*=610 (horizontal axes) are illustrated. The upper panel shows the values of *ϕ*_*i*_ for the subset of clonotypes. (Each *i* has the same value of *ϕ*_*i*_ in each realisation.) In the lower panel, the vertical axis is the realisation number. A blue rectangle at position *i* in realisation *j* indicates that the clonotype has survived to $t=10^{4}$ in that realisation. Many clonotypes never survive; some usually do. The parameter values are γ=10, *M*=1000, *p*=0.1. (For interpretation of the references to color in this figure caption, the reader is referred to the web version of this paper.)

Is the competition selective or stochastic? That is, do some clonotypes survive and other die because they are better suited to the competitive environment, or is it simply a matter of chance? It is possible to answer this question by carrying out a series of independent realisations, each with the same matrix *A*, encoding the pattern of connections between self pMHC and T cell clonotypes. The realisations correspond to independent series of stochastic events occurring to identical in silico individuals. The results of ten such realisations are summarised in Fig. 6. About five per cent of clonotypes survive, which could be achieved in two ways. If survival is determined by chance, any one clonotype will survive any one realisation with the same probability (here, about 0.05). If survival is determined by selection, a small subset of clonotypes will survive in most realisations (De Boer and Perelson, 1997). The presence of several near-complete vertical lines, in the subset of clonotypes shown, indicates that the competition is selective. Note, too, the correlation between higher values of *ϕ*_*i*_ and survival frequency.

**Fig. 7:**
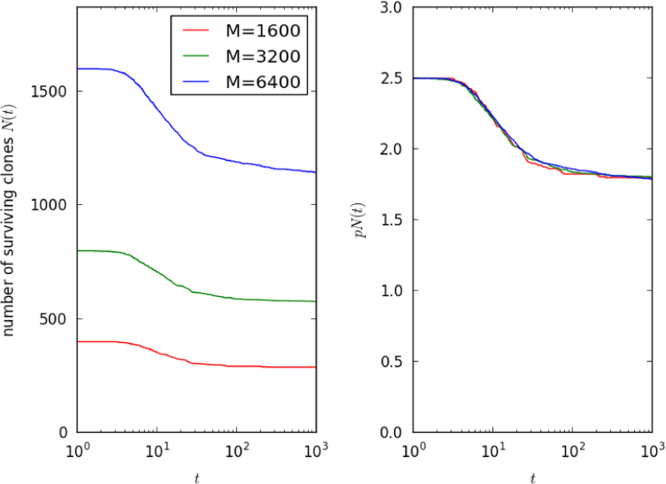
Scaling of the dynamics with the size of the self pMHC environment. Different numerical runs, with *M*=1600, *M*=3200 and *M*=6400, without thymic input. In each case, *pM*=100, μ=1, $N(0)=\frac{1}{4}M$ and γ=10. In the vertical axis on the right, the number of surviving clonotypes, as a function of time, is multiplied by *p*. The product *pM* is the mean number of self pMHC recognised by a single TCR clonotype; the product *pN* is the mean number of clonotypes that recognise a single self pMHC.

While it is straightforward to perform computational studies of competition between hundreds or even thousands of T cell clonotypes, it is more challenging to study physiological numbers of clonotypes. It is therefore important to understand how the system can be scaled in such a way that the dynamics of a large system, viewed from any one clonotype, is independent of system size. In the random-connection case, where any one of *N* T cell clonotypes recognises any one of *M* self pMHCs with probability *p*, the number recognised by one T cell clonotype has a binomial distribution with parameter *pM*. We generate ever-larger systems, maintaining an invariance from the perspective of any one clonotype, by increasing *M* but keeping the product *pM* constant. The result, as illustrated in Fig. 7 with a set of three numerical runs, scales in the sense that the dynamics of the quantity *pN*(*t*) is preserved. (The product *pM* is the mean number of self pMHC recognised by a single TCR clonotype; the product *pN* is the mean number of clonotypes that recognise a single self pMHC.)

### 4.5 Thymic production

**Fig. 8:**
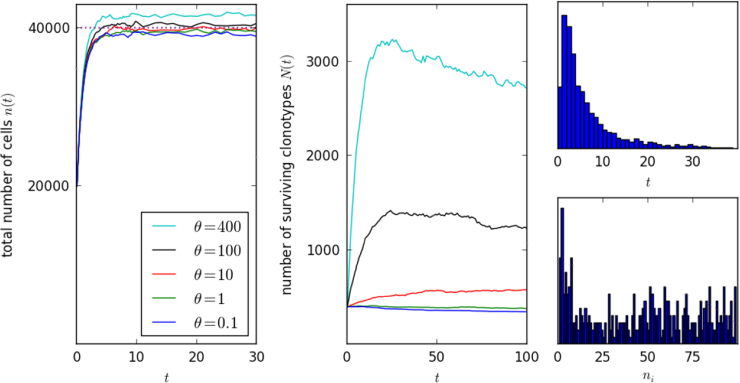
Input of new clonotypes from the thymus, while barely affecting the total number of cells, determines the late-time number of surviving clonotypes. For comparison with adult human homeostasis, time is measured in years. The thymic output rates are θ=0.1 year^−1^ (blue), θ=1 year^−1^ (green), θ=10 year^−1^ (red), θ=100 year^−1^ (black) and θ=400 year^−1^ (cyan). The remaining parameter values are *M*=4000, *pM*=100, γ=10 year^−1^, μ=1 year^−1^, $n_{\theta }=4$ in all cases. The histograms on the right show, for θ=10 year^−1^, the distribution of survival times of clonotypes from the thymus and the distribution of *n*_*i*_(*t*) for surviving clonotypes. (For interpretation of the references to color in this figure caption, the reader is referred to the web version of this paper.)

Three numerical realisations are summarised in Fig. 8. As $\theta n_{\theta }⪡\gamma M$ in all three, thymic production has almost no perceptible effect on the mean total number of cells, *x*(*t*). Even so, it is the strength of thymic production that determines the stationary number of surviving clonotypes. With thymic production included in the system, a bona fide steady state is established, where new clonotypes from the thymus balance those that are driven to extinction. The steady state is established on a long timescale, corresponding to decades in humans. The stationary distribution of clonal sizes (that is, the distribution of numbers of cells in surviving clonotypes) and density of times to extinction, are shown on the right for the case θ=10 year^−1^. In this work we have restricted our attention to homeostasis in adults. In future work we plan to extend the model to consider the full human lifetime, taking into account changes in thymic output and body size during childhood (Bains et al., 2009, Johnson et al., 2012).

**Fig. 9:**
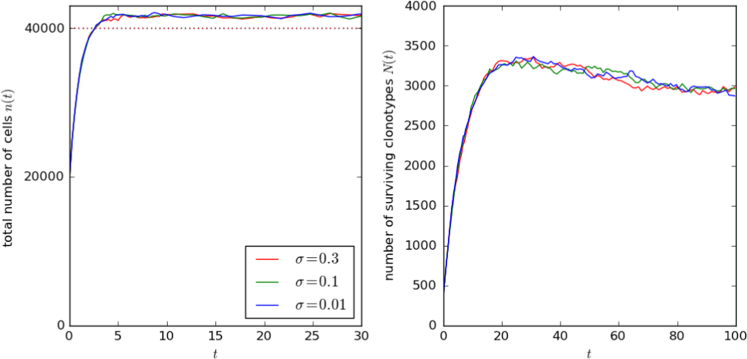
Total number of cells, and total number of surviving clonotypes, as a function of time. Stimulus rates of the self pMHC, *γ*_*q*_, are drawn from a log-normal distribution. The values of *σ* are the ratio of the standard deviation to the mean value of 10 year^−1^. In the case σ=1, the standard deviation is equal to the mean. The remaining parameter values are *M*=4000, *pM*=100, θ=400 year^−1^, μ=1 year^−1^ and $n_{\theta }=4$.

In Fig. 9, we display results from a version of the model with an extra element of structural stochasticity. Instead of a constant stimulus rate, each self pMHC is assigned a strength *γ*_*q*_ drawn from a log-normal distribution. The parameter *σ* is the standard deviation of the distribution, divided by the mean. Even with the widest distribution of rates, there is little discernible effect on the repertoire dynamics.

## Figures and Tables

**Table 1:** Estimated parameter values in humans and mice.

Parameter	**Human**	**Mouse**
*μ*	0.5 year^−1^	1 month^−1^
*γ*	10 year^−1^	10^−4^ month^−1^
*θ*	10^9^ year^−1^	$2.5\times 10^{6}$ month^−1^
*M*	10^10^	10^10^
*p*	10^−6^	10^−6^
$n_{\theta }$	4	4
